# Persistence of the immune response after 4CMenB vaccination, and the response to an additional booster dose in infants, children, adolescents, and young adults

**DOI:** 10.1080/21645515.2019.1627159

**Published:** 2019-07-09

**Authors:** Federico Martinón-Torres, Terry Nolan, Daniela Toneatto, Angelika Banzhoff

**Affiliations:** a Translational Pediatrics and Infectious Diseases Section, Pediatrics Department, Hospital Clínico Universitario de Santiago de Compostela, Santiago de Compostela, Spain; b School of Population and Global Health, The University of Melbourne, and Murdoch Children’s Research Institute, Melbourne, Victoria, Australia; c GSK, Siena, Italy; d GSK, Marburg, Germany

**Keywords:** *Neisseria meningitidis*, antibody persistence, immunogenicity, 4CMenB, meningococcal serogroup B

## Abstract

The multicomponent meningococcal serogroup B vaccine, 4CMenB, has demonstrated effectiveness in preventing invasive MenB disease in infants and in controlling MenB outbreaks. The need for/timing of additional booster doses is not yet established. We reviewed eight studies that evaluated antibody persistence and booster following primary 4CMenB vaccination of infants, children, adolescents, and young adults. Putative seroprotective hSBA titers for ≥1 vaccine antigen were maintained by 76–100% of children 24–36 months after priming during infancy and in 84–100% after priming in the second year of life. hSBA levels were higher in vaccinees at 4 and 7.5 years following priming during adolescence than in vaccine-naïve individuals of a similar age. Antibodies persisted at higher levels to NHBA and NadA than to PorA or fHbp. Booster vaccination induced robust anamnestic responses, demonstrating effective priming by 4CMenB across age-groups. These data can inform decision-making to optimize vaccination strategies.

## Introduction

*Neisseria meningitidis* is one of the most common causes of bacterial meningitis and can cause severe disease with a high risk of permanent sequelae or death.^1,2^ Disease can occur at any age, but infants under one year of age are most at risk, with a secondary peak in invasive meningococcal disease (IMD) incidence during adolescence in some countries.^3^ The six serogroups (A, B, C, W, Y, and X) that cause the majority of IMD vary geographically.^4^ Circulating serogroups also undergo cyclic variation with periodic outbreaks and epidemics of disease as new strains emerge in susceptible populations.^4^ Serogroup predominance also occurs under the influence of meningococcal national immunization programs (NIP).^5^ In many industrialized countries, particularly countries with existing meningococcal serogroup C NIP, serogroup B (MenB) is the most frequent cause of IMD.^6^ In 2016, MenB caused approximately 73% of IMD cases in infants, and more than 50% in 15–24-year olds in Europe,^7^ and 60% of IMD cases in children less than 5 years of age and 50% in 11–23-year olds in the United States (US).^8^ The overall case fatality rate for MenB IMD was 7–11%.^7,8^

The multicomponent meningococcal serogroup B vaccine, 4CMenB (*Bexsero*, GSK) was the first broadly protective MenB vaccine, licensed in the European Union in 2013 and in the US in 2015. 4CMenB contains four antigens: Neisserial Heparin Binding Antigen (NHBA),^9^ factor H binding protein (fHbp),^10^ and Neisseria adhesin A (NadA),^11^ combined with the outer membrane vesicle component of a vaccine obtained from the NZ 98/254 strain (B:4:P1.7–2,4; ST-42 [cc41/44]), which was used to successfully curtail an outbreak in New Zealand.^12^ Of 40 clinical MenB isolates from the United Kingdom (UK) and Wales, 64% expressed fHbp at levels (relative potency) higher than the positive bacterial threshold predicted to indicate susceptibility to bactericidal killing in a serum bactericidal assay using human complement (hSBA).^13^ A total of 55% expressed NHBA and 20% expressed PorA, and only 0.6% expressed NadA. Similar trends have been observed in Europe.^14^ in the US, the percentage of 442 clinical MenB isolates with relative potencies greater than the positive bacterial threshold was 53% for fHbp, 83% for NHBA, 5.9% for PorA and 2.5% for NadA.^15^

A second MenB vaccine, rLP2086 (*Trumenba*, Pfizer) was licensed in the US in 2014, and in the European Union in 2017. rLP2086 is a bivalent vaccine that contains equal amounts of two variants (variants 1 and 3) of fHbp in a lipidated form. The two fHbp variants were selected because of their ability to induce serum bactericidal activity against a range of MenB strains.^16–18^

4CMenB was authorized on the basis of demonstrated safety and immunogenicity in terms of the percentage of subjects who achieved threshold antibody levels hSBA, which is widely accepted as a surrogate marker of protection against meningococcal disease.^19^

In 2017, the first estimate of the effectiveness of two doses of 4CMenB in preventing IMD in infants was 82.9% (95% confidence interval [CI] 24.1–95.2),^20^ substantiating pre-licensure estimates of vaccine coverage and potential impact. As yet the duration of protection is not known. Decisions about the need for and timing of booster doses to maintain circulating antibodies during periods of high risk are informed by antibody persistence data and booster studies in target populations.

The aim of this review is to provide a comprehensive picture of the available data 5 years after the first licensure of 4CMenB. To this end, we bring together data from eight studies (nine cohorts) that assess antibody persistence and the immunogenicity of additional booster doses after priming with 4CMenB, in infants, children, adolescents, and young adults.

**Figure 1.**
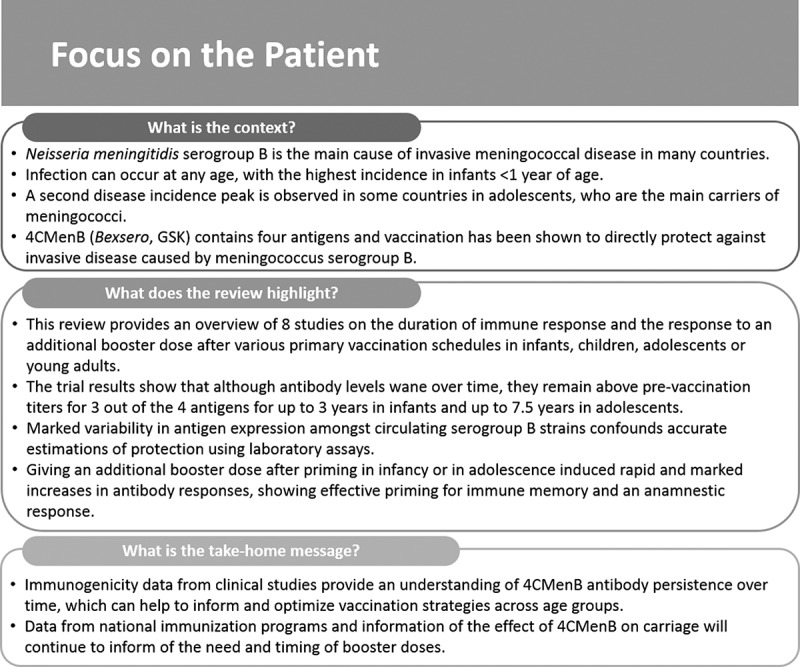
Focus on the patient section.

Figure 1 summarizes the research, clinical relevance, and impact on the patient population.

## Antibody persistence following vaccination with 4CMenB

Antibody persistence after 4CMenB vaccination has been measured in cohorts vaccinated in eight clinical trials (Tables 1 and 2). These studies were conducted in North America, Australia, Chile, and in Europe, and included participants vaccinated in all age groups from 6 weeks of age up to 25 years of age. In infants, antibody persistence was assessed after primary vaccination in 3-dose (2 + 1) and 4-dose (3 + 1) vaccination schedules.^21–28^ In children older than 1 year of age, adolescents, and young adults, persistence was assessed after receiving two priming doses at an interval of at least 1 month. Studies/groups that assessed persistence after three-dose priming in children adolescents and young adults are not included here because the recommended schedule for these age groups is two doses.

**Table 1.** Clinical studies assessing antibody persistence following 4CMenB vaccination in infants.

				% with hSBA ≥ 4 (95% CI)				hSBA GMT (95% CI)			
Study number, country (identifier*)	Priming schedule	N	Months post last vaccination	fHbp	NadA	PorA	NHBA	fHbp	NadA	PorA	NHBA
Study 1, Europe, UK NCT01717638 Iro et al, 2017^22^	2,4,6m, 12 or 18 or 24m booster	58-60	24m	24	97	12	74	2.41	69	1.38	9.08
				(14-37)	(88-100)	(5-23)	(61-85)	(1.83-3.19)	(50-96)	(1.11-1.72)	(5.97-14)
		59-60	30m	18	98	8	68	1.68	69	1.29	7.36
				(10-30)	(91-100)	(3-18)	(54-79)	(1.29-2.19)	(50-94)	(1.05-1.59)	(4.94-11)
		65-67	36m	12	93	9	54	1.75	36	1.25	6.14
				(5-22)	(83-98)	(2-18)	(41-66)	(1.36-2.25)	(27-48)	(1.03-1.52)	(4.19-8.99)
	2,3,4m + 12 or 18 or 24m booster	28	24m	21	96	11	75	2.2	101	1.62	11
				(8-41)	(82-100)	(2-28)	(55-89)	(1.37-3.53)	(57-177)	(1.21-2.16)	(5.9-21)
		28	30m	25	89	11	75	2.2	62	1.25	11
				(11-45)	(72-98)	(2-28)	(55-89)	(1.38-3.49)	(36-108)	(0.94-1.66)	(5.92-20)
		40-42	36m	12	90	10	68	1.51	52	1.32	9.61
				(4-26)	(77-97)	(3-23)	(51-81)	(1.04-2.18)	(34-81)	(1.05-1.65)	(5.81-16)
Study 2, Europe NCT00847145, NCT01139021 Vesikari et al, 2013^23^ and 2015^24^	2,4,6, 12m+ MMRV at 12m or 13m	305	12m	62	97	17	36	6.5	81	1.9	3.4
								(5.6-7.5)	(71-94)	(1.7-2.2)	(2.9-3.9)
Study 3, UK NCT01027351 Snape et al, 2013^25^, McQuaid et al 2015^26^	2,4,6, 12m	15-17	28m	65	76	41	67	5.3	28	2.8	5.3
				(38-86)	(50-93)	(18-67)	(38-88)	(3.3-8.8)	(9.4-83)	(1.4-5.6)	(2.3-12)
	2,4,6, 12, 40m	16	20m	44	88	69	88	4.68	136	4.95	10
				(20-70)	(62-98)	(41-89)	(62-98)	(2.3-9.52)	(51-365)	(3.54-6.92)	(5.67-19)
Study 4, UK NCT01026974 Snape et al 2013^27^, McQuaid et al 2014^28^	6,8, 12m	14	28m	36	100	14	79	2.55	29	1.74	7.11
				(13-65)	(77-100)	(2-43)	(49-95)	(1.15-5.66)	(18-47)	(0.91-3.33)	(3.61-14)
	6,8, 12, 40m	11-12	20m	67	100	17	45	4.69	119	1.63	5.51
				(35-90)	(72-100)	(2-48)	(17-77)	(1.98-11)	(56-252)	(0.86-3.08)	(2.19-14)
Study 5, Europe NCT01894919 Martinon-Torres et al, 2017^21^	2.5, 3.5, 5, 11m	127-140	24-36m	51	84	45	36	4.17	44	3.48	2.77
				(42.8-60.0)	(77.2-89.9)	(36.6-53.6)	(27.9-45.2)	(3.40-5.13)	(32-60)	(2.78-4.36)	(2.06-3.71)
	3.5, 5, 11m	111-131	24-36m	53	88	38	38	4.48	52	2.98	3.03
				(43.8-61.5)	(80.9-92.9)	(29.8-47.1)	(28.8-47.5)	(3.60-5.57)	(37-72)	(2.35-3.78)	(2.20-4.16)
	6, 8, 11m	109-119	24-36m	61	93	56	45	5.62	83	4.86	3.17
				(52.0-70.1)	(87.2-97.1)	(46.9-65.4)	(35.4-54.8)	(4.48-7.04)	(58-117)	(3.80-6.22)	(2.30-4.38)

*identifier in www.clinicaltrials.gov, N = number of subjects in the persistence cohorts, CI = confidence interval, hSBA = serum bactericidal assay using human complement source, GMT = geometric mean antibody titer, m = months, UK = United Kingdom, fHbp = factor H binding protein, NadA = Neisseria adhesin A, PorA = porin A, NHBA = Neisserial Heparin Binding Antigen.

**Table 2.** Clinical studies assessing antibody persistence following 4CMenB vaccination in toddlers, children, adolescents and young adults.

				% with hSBA ≥ 4* (95% CI)				hSBA GMT (95% CI)			
Study number, (identifier*)	Schedule	N	Time post-last vaccination	fHbp	NadA	PorA	NHBA	fHbp	NadA	PorA	NHBA
Study 2, Europe NCT00847145, NCT01139021, Vesikari et al, 2015 ^24^	2 doses at 13,15m	64-67	12m	75	97	18	39	14	73	1.7	3.7
								(9.0-23)	(43-123)	(1.1-2.6)	(2.1-6.5)
	2 doses at 12,14m	18-19	12m	56	94	6	28	8.4	71	1.2	3.3
	2 doses at 24,26m	104-116	6m	93	96	18	-	22	71	1.9	8.0
Study 1, Europe, UK NCT00721396, Sadarangani et al, 2017^31^	2 doses at 12,14m or 18,20m or 24,26m	123	22-34m	9-11	84-100	0-18	59-60	<5	≥5	<5	≥5
Study 5, Europe NCT01894919, Martinon-Torres et al, 2017^21^	2 doses at 2-5yrs	65-68	24-36m	52	79	29	42	3.97	21	2.81	3.53
				(39.7-64.6)	(67.4-88.1)	(19.0-41.7)	(29.4-54.4)	(2.99-5.28)	(14-33)	(2.07-3.82)	(2.38-5.25)
	2 doses at 6-10yrs	173-178	24-36m	58	85	50	66	5.75	21	4.57	7.82
				(50.3-65.2)	(79.4-90.3)	(42.2-57.3)	(58.9-73.5)	(4.78-6.91)	(16-28)	(3.74-5.59)	(6.04-10)
Study 6, Australia, Canada NCT02446743, Nolan et al, 2017^35,39^ **	2 doses at 11-17yrs	134-144	4 yrs	30	84	9	75	2.43	24	1.31	13
				(22.5-38.0)	(77-90)	(4.9-14.9)	(66.9-81.7)	(2.04-2.89)	(19-30)	(1.17-1.45)	(9.86-18)
Chile NCT01148524 Santolaya et al, 2013^33,35^ **	2 doses 11-17yrs	106	18-24m	82	94	77	-	31	46	20	-
				(77-87)	(91-97)	(71-82)		(24-39)	(38-55)	(16-24)	
	2 doses at 11-17yrs	120-131	7.5 yrs	44	84	29	81	4.51	31	2.56	22
				(35.6-53.2)	(76.4-90.2)	(21.1-37.3)	(73.1-87.3)	(3.57-5.69)	(23-42)	(2.07-3.17)	(16-29)
Study 7 UK NCT01214850 Read et al, 2017 ^36^	2 doses at 18-24yrs	192	11m	95	97	85	-	68	55	34	-
				(91-98)	(94-99)	(79-90)		(54-84)	(45-67)	(26-45)	
Study 8 US, Poland Szenborn et al, 2018^37^	2 doses at 10-25yrs	36	2 yrs	34	94	16	50	39	3.22	1.75	4.56
				(19.6-51.4)	(81.3-99.3)	(6.0-31.3)	(33.4-66.6)	(25-62)	(2.18-4.76)	(1.24-2.47)	(2.97-7.01)

*identifier in www.clinicaltrials.gov, N = number of subjects in the persistence cohorts, CI = confidence interval, hSBA = serum bactericidal assay using human complement source, GMT = geometric mean antibody titer, m = months, yrs = years, UK = United Kingdom, US = United States, fHbp = factor H binding protein, NadA = Neisseria adhesin A, PorA = porin A, NHBA = Neisserial Heparin Binding Antigen. *The pre-specified cut-off was 5 for all antigens in Study 8. Immune responses for NHBA were determined using strain M10713 in all studies except Study 8, which used strain M07-0241084.

4CMenB elicits bactericidal antibodies against the four key antigens combined in the vaccine.^29^ Immunogenicity of each component is measured by antigen-specific hSBA using *N. meningitidis* strains selectively recognized by antibodies against each of the antigens. The ‘indicator’ strains were all isolated from cases of IMD, and each strain only measures the contribution of one antigen in the hSBA assay, thereby providing evidence of the functional antibody response to the individual vaccine components.^29^ Immune responses to fHbp, NadA, and Porin A (PorA) were determined using the indicator strains H44/76, 5/99, and NZ98/254, respectively. When tested, immune responses for NHBA were determined using strain M10713 in all studies except Study 8, which used strain M07-0241084. All assays were performed in the same laboratory (GSK, Marburg, Germany) using the same quality control procedures, with the exception of Studies 5 and 7. For these studies, testing of NadA, fHbp, and PorA was done at Public Health England Laboratory, Manchester, UK, a national reference laboratory. Here we report the percentage of participants who maintained an hSBA titer of at least 4 (an hSBA cut-off of 5 was used in studies 1, 2, and 8).

### Infants

Antibody persistence following vaccination in infancy was measured in five studies for up to 36 months after the last 4CMenB dose (Table 1).^21–28^

In Study 1, infants received three doses of 4CMenB at either 2, 4, 6 months or 2, 3, 4 months of age concomitantly with routine vaccines (diphtheria-tetanus-acellular pertussis-hepatitis B-inactivated polio and *Haemophilus influenzae* type b vaccine, and 7-valent pneumococcal conjugate vaccine),^30^ and received a fourth dose of 4CMenB 6, 12, or 18 months later. Antibody persistence was assessed 24 to 36 months after the fourth dose.^22^ The results show a progressive decrease in hSBA antibodies over time after the fourth dose (Table 1). By 36 months after the fourth dose, the percentage of children with hSBA titers ≥4 was 90% to 93% for NadA, 54% to 68% for NHBA, 12% for fHbp, and 9% to 10% for PorA.^22^

In Study 2, infants received 4CMenB at 2, 4, and 6 months with a fourth dose administered with or without measles-mumps-rubella-varicella vaccine at 12 months of age.^23,24^ One year after the fourth dose, the percentage of children with hSBA titers ≥4 was 97% for NadA, 62% for fHbp, 36% for NHBA, and 17% for PorA.^24^

Two small studies (Study 3^25,26^ and Study 4^27,28)^ assessed antibody persistence after 4-dose (3 + 1 starting at 2 months of age with booster at 12 months) and 3-dose (2 + 1 starting at 6 months of age with booster at 12 months) priming, respectively. At 40 months of age, the percentage of children with hSBA titers ≥4 was 36% to 65% for fHbp, 76% to 100% for NadA, 67% to 79% for NHBA, and 14% to 41% for PorA. An additional booster dose of 4CMenB was administered at age 40 months, with evaluation of antibody persistence 20 months later when the children were 5 years old. At 5 years of age, the percentage of children with hSBA titers ≥4 was 44% to 67% for fHbp, 88% to 100% for NadA, 17% to 69% for PorA, and 45% to 88% for NHBA.^25–28^ The small number of subjects may account for some of the variability in these results, but overall, the results suggest similar kinetics of the antibody response after booster vaccination at 12 months or at 40 months of age.

**Figure 2.**
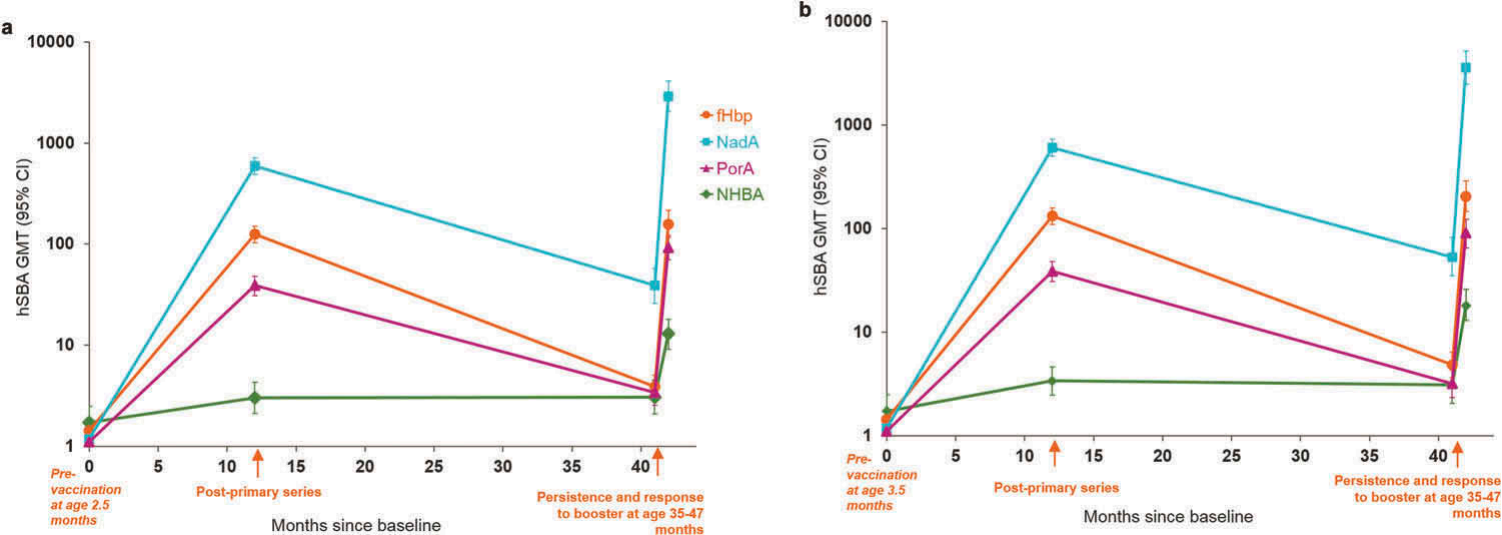
hSBA GMTs one month following priming and booster at 35–47 months of age: (a) 3 + 1 schedule (2.5, 3.5, 5 and 11 months of age), (b) 2 + 1 schedule (3.5, 5 and 11 months of age) (Study 5). Footnotes: Sample size: A 127–140 in the booster cohort; B 111–131 in the booster cohort; CI = confidence interval, hSBA = serum bactericidal assay using human complement source, GMT = geometric mean antibody titer, fHbp = factor H binding protein, NadA = Neisseria adhesin A, PorA = porin A, NHBA = Neisserial Heparin Binding Antigen.

Study 5 assessed antibody persistence 24 to 36 months after either four doses at 2.5, 3.5, 5, and 11 months of age (3 + 1) or three doses at 3.5, 5, and 11 months of age (2 + 1).^21^ At 24 to 36 months after the last dose administered, persisting hSBA titers ≥4 and geometric mean titers (GMTs) for each of the indicator strains were similar in children who had received a 3 + 1 or 2 + 1 primary vaccination schedule (Figure 2(a,b)). The percentage of children with hSBA titers ≥4 was 51% in the 3 + 1 dose group versus 53% in the 2 + 1 group for fHbp, 84% versus 88% for NadA, 45% versus 38% for PorA, and 36% versus 38% for NHBA. Antibody persistence following vaccination of infants at 6, 8, and 11 months of age was assessed in the same study. At 24 to 36 months after the last dose administered, the percentage of children with hSBA titers ≥4 was 61% for fHbp, 93% for NadA, 56% for PorA, and 45% for NHBA.

### Children aged 1 to 2 years of age

Two studies assessed antibody persistence after two-dose vaccination of toddlers between 12 and 23 months of age with 4CMenB (Table 2).^24,31^

Twenty-two to 34 months after two 4CMenB doses administered at 12 and 14 months, 18 and 20 months, or 24 and 26 months in Study 1, antibodies to fHbp had reduced markedly (9% to 11% had hSBA titers ≥4 for fHbp) and 0% to 18% had hSBA titers ≥4 for PorA,^31^ whereas as observed in infants, antibodies to NadA and NHBA remained at higher levels (84% to 100% with hSBA titers ≥4 for NadA, and 59% to 60% for NHBA).

In Study 2, toddlers were vaccinated with two doses of 4CMenB at 13 and 15 months, or at 12 and 14 months of age. Twelve months after vaccination, the percentage of children with hSBA titers ≥4 was 94% to 97% for NadA and 6% to 18% for PorA, 56% to 75% for fHbp, 28% to 39% for NHBA.^24^ In the same study, children aged 2 years of age received two doses of 4CMenB at 24 and 26 months of age. Six months after the second dose, a high percentage of children continued to have hSBA titers ≥4 antibodies to fHbp and NadA (93% to 96%) while 18% had hSBA titers ≥4 for PorA.

### Children older than 2 years and adolescents

One study (Study 5) assessed antibody persistence 24 to 26 months after two priming doses of 4CMenB administered to children between 2 and 10 years of age.^21^ The percentage of children with hSBA titers ≥4 24 to 36 months after the second dose was 52% to 58% for fHbp, 79% to 85% for NadA, 29% to 50% for PorA, and 42% to 66% for NHBA.^21^

Study 6 assessed antibody persistence in adolescents who received two doses of 4CMenB between 11 and 17 years of age in two parent studies.^32,33^ In the cohort of subjects enrolled in Canada and Australia who were assessed 4 years after vaccination, the percentage of subjects with hSBA titers ≥4 had declined markedly for fHbp and PorA (30% and 9%, respectively), but remained at high levels for the other antigens (84% for NadA and 75% for NHBA). In subjects enrolled in Chile, antibodies to three indicator strains (NHBA not tested) remained high (77% to 94% with hSBA titers ≥4) 18–24 months after vaccination.^33^ At 7.5 years after vaccination, the percentage of vaccinees with hSBA titers ≥4 was 84% for NadA, 81% for NHBA, 44% for fHbp, and 29% for PorA.^34^ In a cohort of vaccine-naïve subjects of a similar age, the percentage with hSBA titers ≥4 was 13% for fHbp, 24% for NadA, 14% for PorA, and 79% for NHBA.^35^

### Young adults

Study 7 assessed antibody persistence in UK university students vaccinated with two 4CMenB doses at 18 to 24 years of age.^36^ Eleven months post-vaccination, a high percentage of participants (85% to 97%) maintained hSBA titers ≥4 against three indicator strains (NHBA not tested) (Table 2). GMTs for each test strain remained higher than pre-vaccination levels.^36^

Study 8 assessed antibody persistence 2 years after vaccination with two 4CMenB doses at 10–25 years of age.^37^ The percentage of participants with hSBA titers ≥4 at 2 years post-vaccination was 34% for fHbp, 94% for NadA, 16% for PorA, and 50% for NHBA (Table 2). hSBA GMTs for fHbp, NadA, and PorA decreased by year 2 but remained higher than pre-vaccination levels.

## Evidence of immune memory – response to an additional dose of 4CMenB

Booster vaccination with 4CMenB is recommended in all countries after primary vaccination during infancy. In Europe, a booster dose in all children who receive initial vaccination (two doses or three doses) before 2 years of age is additionally recommended.^38^ The need for additional doses in children who are primed after 2 years of age or during adolescence has yet not been established.

Five studies reported on the immunogenicity and safety of an additional booster dose of 4CMenB (Table 3).^21,22,25,27,39^ Four studies assessed a pre-school booster administered at 3 to 4 years of age.^21,22,25,27^ Study 5 assessed a booster dose after vaccination during childhood at age 2 to 10 years,^21^ and Study 6 assessed booster vaccination of adolescents and young adults 4,^39^ or 7.5 years after initial vaccination at 11 to 17 years of age.^35^

**Table 3.** Clinical studies assessing booster doses of 4CMenB.

					% with hSBA ≥ 4 (95% CI)				hSBA GMT (95% CI)			
Study number, country (identifier*)	Priming schedule	Age at booster	N	Time point	fHbp	NadA	PorA	NHBA	fHbp	NadA	PorA	NHBA
Study 5, Europe NCT01894919 Martinon-Torres et al, 2017^21^	2.5, 3.5, 5, 11m	35-47m	78-92	Pre	48	84	45	38	3.91	39	3.41	3.05
					(37.3-58.5)	(74.5-90.6)	(34.2-55.3)	(27.7-50.2)	(3.01-5.08)	(26–58)	(2.57-4.54)	(2.08-4.46)
			88-96	Post	99	99	99	75	158	2908	92	13
					(94.3-99.97)	(94.3-99.97)	(94.3-99.97)	(64.6-83.6)	(116-215)	(2059-4107)	(70-122)	(9.15-18)
	3.5, 5, 11m	35-47m	68-87	Pre	51	91	42	37	4.84	53	3.17	3.10
					(40.1-62.1)	(82.7-95.9)	(31.3-53.0)	(25.4-49.3)	(3.66-6.41)	(35-82)	(2.34-4.31)	(2.05-4.68)
			79-87	Post	100	99	100	84	205	3593	91	18
					(95.8-100)	(93.8-99.97)	(95.8-100)	(73.5-90.9)	(147-287)	(2474-5218)	(68-123)	(13-26)
	6, 8, 11m	35-47m	65-76	Pre	64	95	52	49	6.21	89	4.86	3.58
					(52.1-74.8)	(87.1-98.5)	(40.2-63.7)	(36.6-61.9)	(4.65-8.31)	(57-139)	(3.54-6.67)	(2.35-5.45)
			67-76	Post	100	97	100	97	288	3677	133	40
					(95.2-100)	(90.8-99.68)	(95.2-100)	(89.6-99.64)	(204-408)	(2495-5419)	(97-181)	(27-59)
	2 doses at 2-5yrs	4-7yrs	39-32	Pre	39	74	25	28	3.14	19	2.99	2.31
					(21.8-57.8)	(55.4-88.1)	(11.5-43.4)	(12.7-47.2)	(2.08-4.75)	(9.92-35)	(1.92-4.65)	(1.29-4.13)
			30-32	Post	97	100	100	93	155	3205	71	32
					(83.8-99.9)	(89.1-100)	(89.1-100)	(77.9-99.2)	(95-252)	(1860-5526)	(46-110)	(19-54)
	2 doses at 6-10yrs	8-12yrs	87-91	Pre	59	86	47	69	6.15	22	4.49	7.83
					(48.5-69.5)	(76.8-92.2)	(36.7-58.0)	(58.1-78.5)	(4.77-7.93)	(15-32)	(3.40-5.92)	(5.52-11)
			89-91	Post	99	100	100	96	258	2921	82	53
					(94.0-99.97)	(96.0-100)	(96.0-100)	(88.9-98.8)	(190-349)	(2079-4104)	(63-108)	(38-73)
Study 1, Europe, UK NCT00721396 Iro et al, 2017^22^	2, 4, 6, 12m	4yrs	65-67	Pre	12	93	9	54	1.75	36	1.25	6.14
					(5-22)	(83-98)	(2-18)	(41-66)	(1.36-2.25)	(27-48)	(1.03-1.52)	(4.19-8.99)
			25-26	Post	100	100	92	84	109	766	21	22
					(87-100%)	(87-100%)	(75-99)	(64-95)	(68-175)	(492-1192)	(13-34)	(14-35)
	2, 4, 6, 18m	4yrs	59-60	Pre	18	98	8	68	1.68	69	1.29	7.36
					(10-30)	(91-100)	(3-18)	(54-79)	(1.29-2.19)	(50-94)	(1.05-1.59)	(4.94-11)
			18	Post	100	100	83	89	119	1725	11	21
					(81-100)	(81-100)	(59-96)	(65-99)	(67-211)	(1013-2936)	(6-19)	(12-35)
	2, 4, 6, 24m	4yrs	58-60	Pre	24	97	12	74	2.41	69	1.38	9.08
					(14-37)	(88-100)	(5-23)	(61-85)	(1.83-3.19)	(50-96)	(1.11-1.72)	(5.97-14)
			16	Post	100	100	94	88	112	923	30	31
					(79-100)	(79-100)	(70-100)	(62-98)	(61-208)	(520-1637)	(16-55)	(17-55)
	2, 3, 4, 12m	4yrs	40-42	Pre	12	90	10	68	1.51	52	1.32	9.61
					(4-26)	(77-97)	(3-23)	(51-81)	(1.04-2.18)	(34-81)	(1.05-1.65)	(5.81-16)
			36-40	Post	97	100	95	97	159	1668	26	51
					(87-100)	(91-100)	(83-99)	(85-100)	(108-235)	(1155-2408)	(18-38)	(35-75)
	2, 3, 4, 18m	4yrs	28	Pre	25	89	11	75	2.2	62	1.25	11
					(11-45)	(72-98)	(2-28)	(55-89)	(1.38-3.49)	(36-108)	(0.94-1.66)	(5.92-20)
			25-26	Post	100	100	92	100	135	1227	18	51
					(87-100)	(87-100)	(75-99)	(86-100)	(83-219)	(783-1921)	(11-28)	(32-81)
	2, 3, 4, 24m	4yrs	28	Pre	21	96	11	75	2.2	101	1.62	11
					(8-41)	(82-100)	(2-28)	(55-89)	(1.37-3.53)	(57-177)	(1.21-2.16)	(5.9-21)
			25-26	Post	100	100	92	100	120	1252	26	44
					(87-100)	(87-100)	(75-99)	(86-100)	(74-195)	(795-1972)	(16-42)	(27-70)
Sadarangani et al, 2017^31^	2 doses at 12, 14m or 18, 20m or 24, 26m	4yrs	123	Pre	9-11	84-100	0-18	59-60	<5	≥5	<5	≥5
			119	Post	100	100	70-100	90-100	>100	>1000	>10	>10
Study 3, UK NCT01027351 Snape et al, 2013^25^	2,4,6, 12m	3.5yrs	15-17	Pre	65	76	41	67	5.3	28	2.8	5.3
					(38-86)	(50-93)	(18-67)	(38-88)	(3.3-8.8)	(9.4-83)	(1.4-5.6)	(2.3-12)
			18-19	Post	100	100	89	94	89	1708	47	39
					(82-100)	(81-100)	(67-99)	(73-100)	(68-116)	(774-3771)	(20-107)	(22-69)
Study 4, UK NCT01026974 Snape et al 2013 ^27^	6, 8, 12m	3.5yrs	14	Pre	36	100	14	79	2.55	29	1.74	7.11
					(13-65)	(77-100)	(2-43)	(49-95)	(1.15-5.66)	(18-47)	(0.91-3.33)	(3.61-14)
			14	Post	100	100	93	93	114	926	32	23
					(77-100)	(77-100)	(66-100)	(66-100)	(59-222)	(432-1988)	(14-71)	(13-41)
Study 6, Australia, Canada NCT02446743, Nolan et al, 2017^35,39^	2 doses at 11-17yrs	15-22yrs	118-142	Pre	30	82	9	75	2.38	22	1.32	13
					(22.4-38.1)	(74.1-88.6)	(5.0-15.1)	(67.0-81.9)	(1.94-2.93)	(16-30)	(1.15-1.50)	(9.99-17.0)
			124-142	Post	98	100	94	99	162	2421	29	65
					(93.9-99.6)	(97.1-100)	(89.2-97.5)	(96.1-99.9)	(132-198)	(1981-2959)	(24-36)	(55-76)
Chile	2 doses at 11-17yrs	18-24yrs	93-127	Pre	46	84	28	80	4.65	31	2.48	22
					(36.8-54.7)	(74.8-90.7)	(20.1-37.0)	(72.3-86.8)	(3.45-6.27)	(21-44)	(1.90-3.23)	(16-29)
			102-127	Post	100	100	93	99	269	1951	41	113
					(97.1-100)	(96.4-100)	(87.3-97.1)	(95.7-100)	(223-325)	(1629-2337)	(32-52)	(92-138)

*identifier in www.clinicaltrials.gov,

N = number of subjects in the persistence cohorts, CI = confidence interval, hSBA = serum bactericidal assay using human complement source, GMT = geometric mean antibody titer, m = months, yrs = years, UK = United Kingdom, fHbp = factor H binding protein, NadA = Neisseria adhesin A, PorA = porin A, NHBA = Neisserial Heparin Binding Antigen, pre = at the time of the booster dose, post = one month after the booster.

4CMenB elicited booster responses in pre-school children primed with 4CMenB in infancy, with marked increases in hSBA GMTs and in the percentage of children with hSBA titers ≥4 after the booster dose compared to the pre-booster time point (Table 3). Post-booster immune responses were observed for all four vaccine antigens in children who had received three or four prior doses of 4CMenB during infancy (Figure 2).^21^ In Study 1, fourfold increases in hSBA titers post-booster were observed in 85% to 100% of participants for NadA, 92% to 100% for fHbp, 61% to 88% for PorA and 31% to 49% for NHBA.^22^ In two studies, geometric mean ratios of hSBA titers (post divided by pre-booster GMT) ranged from 3.25 to 70 for each vaccine antigen.^25,27^ Across the four booster studies conducted in this age group, the percentage of children with hSBA titers ≥4 after the booster dose was 97% to 100% for fHbp and NadA, 83% to 100% for PorA, and 75% to 100% for NHBA.^21,22,25,27^

In children who received a booster dose of 4CMenB at 4 to 12 years of age after priming between age 2 to 10 years, the percentage with hSBA titers ≥4 post-booster was also high, ranging from 93% to 100% for each of the four indicator strains.^21^

**Figure 3.**
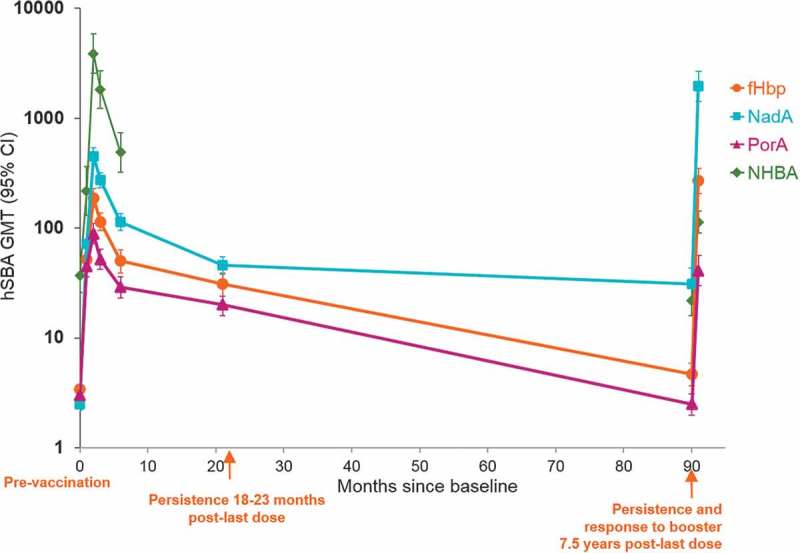
hSBA GMTs one month following priming and booster 7.5 years after two dose priming of adolescents at 11–17 years of age* (Study 6). Footnotes: * Two-dose schedule: 0–1, 0–2, or 0–6 months. Data from baseline through 6 months is from the 0–1 month schedule group; Persistence data from 18–23 months onwards is from pooled 0–1, 0–2, and 0–6 month schedules; Sample size = 93–127 in the pooled schedule booster cohort; CI = confidence interval, hSBA = serum bactericidal assay using human complement source, GMT = geometric mean antibody titer, fHbp = factor H binding protein, NadA = Neisseria adhesin A, PorA = porin A, NHBA = Neisserial Heparin Binding Antigen.

Adolescents aged 11–17 years primed with two 4CMenB doses were administered a booster dose 4 years later (Canadian and Australian cohort) or 7.5 years later (Chilean cohort).^35^ By 7 days after the booster dose at year 4 or year 7.5, 73% to 100% of all subjects had hSBA titers ≥4 for each of the indicator strains, and hSBA GMTs had increased by 33 to 98-fold for fHbp and NadA, and by 3.1 to 7.6-fold for PorA and NHBA. At both time-points, post-booster hSBA GMTs for each vaccine antigen were significantly higher one month after the booster dose than one month after a two-dose catch-up series in subjects of a similar age (manuscript submitted).^35^ Kinetics of the immune response over the 7.5-year period in Chilean participants is shown in Figure 3.

In all age-groups studied, a booster dose of 4CMenB elicited marked increases in the GMTs to each of the four indicator strains that were higher than observed after vaccination of vaccine-naïve subjects of a similar age, consistent with successful priming and an anamnestic response to further exposure. Examination of the kinetics of the booster response in young adults primed during adolescence showed rapid increases in hSBA antibody levels within 7 days post-booster, illustrating a potential benefit to these individuals in the event of an outbreak.^35^

## The clinical implications of waning immunity

For serogroups A, C, W, and Y, antibodies directed against the polysaccharide capsule correlate well with effectiveness.^40^ For MenB, these antibodies are directed against subcapsular antigens, and we still do not know how well they correlate with long-term protection for either of the available broadly protective MenB vaccines.

All MenB IMD strains contain genes for at least one 4CMenB component, and the majority have genes for more than one, although their sequence, surface expression and susceptibility to specific antibody-mediated killing vary widely, potentially providing numerous targets for antibodies induced by vaccination.^41^ Vaccine-induced antibodies to any one of the individual vaccine antigens may be sufficient to confer protection against an invasive organism, depending upon the type and amount of antigen the bacterium expresses. This means that subjects vaccinated with 4CMenB are unlikely to need to maintain high levels of antibodies to all four antigens in order to benefit from some degree of protection. For example, 86% of clinical MenB isolates from US, and 52.5% of UK isolates were targeted by one or two vaccine antigens, most frequently NHBA and/or fHbp.^13,15^

In infants, at least 76% of vaccines maintained putative seroprotective hSBA antibodies to at least one 4CMenB vaccine antigen up to 24–36 months after vaccination. The percentage of infants with persisting antibodies to fHbp and NHBA, which are the vaccines antigens expressed most frequently by clinical isolates from the UK and US, was 12% to 65% and 38% to 79%, respectively.

Only one study (Study 1) assessed antibody persistence more than 12 months after primary vaccination during the second year of life. The available data suggest high antibody persistence until 12 months, with rapid decay of antibodies to fHbp and PorA during the second to third years, similar to antibody decay observed in infants. Antibody persistence for all four vaccine antigens was higher when subjects received primary vaccination as children.

In adolescents, the majority (>80%) of subjects vaccinated with 4CMenB maintained seroprotective hSBA antibodies to at least one 4CMenB vaccine antigen from year 4 through year 7.5. There were 44% of adolescents with persisting antibodies to fHbp and 81% for NHBA at 7.5 years. Interpretation of long-term antibody persistence data can be clouded by the potential boosting effects of natural exposure to the bacterium or to cross-reacting antigens. Assessment of antibody levels in unvaccinated controls can provide some insight into the level of natural exposure in the population. In unvaccinated Chilean subjects of approximately 21 years of age, putative hSBA seroprotection rates ranged between 13% and 79%, suggesting exposure of this population to MenB. Seroprotection rates after 7.5 years in vaccinated subjects were higher for each antigen (range 29% to 84%), and the response to a booster dose was markedly higher than the response of vaccine-naïve subjects a 4CMenB dose, suggesting a long-term benefit from vaccination. The reasons underlying the high persistence rate at 7.5 years in Chilean subjects compared to studies conducted in other countries with shorter follow-up are not understood. However, compared to other countries, high immune responses have been observed previously in Chilean subjects for other vaccines.^42,43^ Potential contributing factors could include different environmental or behavioral exposures to the pathogen or cross-reacting bacteria, for example, through household crowding or smoking.

The body of evidence supporting antibody persistence after primary vaccination with 4CMenB is substantial and encompasses age groups from infancy to early adulthood. Interpretation of the data could be limited by the heterogeneity of the study populations which differed in terms of vaccine schedule and period of long-term follow-up. Baseline titers also differed between populations and the impact of baseline antibodies on persistence and booster was not explored here. Nevertheless, there were marked increases in hSBA GMTs after booster for vaccine antigens where the level of seroprotection prior to the booster dose was already high (NHBA and NadA).

Expression of the different proteins contained in 4CMenB varies markedly among clinical strains and so the presence of seroprotective hSBA antibodies to individual 4CMenB vaccine antigens does not necessarily translate into protection at the individual strain level. However, evidence suggests that the fHbp, NadA, and NHBA antigens in 4CMenB each contain multiple protective epitopes that induce antibody responses.^44^ These antibodies work synergistically to enhance the bactericidal response to each antigen. Vu et al., 2011^45^ showed that antibodies that are non-bactericidal against specific antigens (such as fHbp and NHBA) may become bactericidal when combined. Together these findings suggest that the percentage of subjects who achieve threshold hSBA titers against individual indicator strains may underestimate the level of protection provided due to synergistic effects of multiple antibodies *in vivo*.^13^

Because the mechanics of the protective immune response induced by 4CMenB are complex, predictions around long-term protection are difficult to make. The studies presented here suggest that immunity to PorA wanes rapidly and that antibodies to fHbp also decline markedly between the first and second year post-vaccination. Antibodies to NadA remain high over time in all age groups, suggesting either a different kinetic of this antibody response, or natural exposure to cross-reacting bacteria. The magnitude of the response to NHBA after vaccination is variable, but antibodies appear to persist longer than for PorA or fHbp. Despite declines over time, antibody levels to all indicator strains remain higher in vaccinated subjects compared to vaccine-naïve subjects of the same age.

Surveillance of IMD disease among vaccinated cohorts and possibly examination of the immune status of individuals who experience IMD in spite of partial or full vaccination will provide important insights into the mechanism for long-term protection after 4CMenB vaccination. Importantly, booster vaccination with 4CMenB induced robust immune responses indicating immune memory. Appropriately timed booster doses could be used to restore circulating antibodies during periods of higher disease risk, such as during early childhood up to 5 years of age, and adolescence.

## Next steps: vaccine effectiveness, correlation of immune response and long-term protection, and herd-protection

The clinical significance of waning antibodies is not yet known, but evidence of synergistic effects of antibodies to multiple epitopes on individual antigens contained in 4CMenB suggest that hSBA titers to individual indicator strains may underestimate the level of protection provided.

Data demonstrating 4CMenB effectiveness are now available. 4CMenB was introduced into the UK NIP program for infants in 2015. After 10 months of use, the number of MenB IMD cases decreased from an average of 74 in the previous 4 years to 37 in 2015–16.^20^ The estimated effectiveness of 4CMenB against MenB IMD was 82.9% (95% CI 24.1–95.2),^20^ with second-year estimates of effectiveness reported to be similar to the first year.^46^ An interesting secondary observation has been a concurrent decrease in IMD due to serogroup W (MenW) in infants targeted for 4CMenB vaccination, possibly due to bactericidal activity of 4CMenB against the UK MenW strain.^47^

4CMenB has also been used successfully in campaigns to curtail MenB outbreaks in the Saguenay Lac Saint Jean region of Quebec,^48,49^ and in several universities in the US.^50–52^ In Saguenay Lac Saint Jean, approximatively 49,000 individuals aged 2 months to 20 years (or 82% of the population in this age group) were vaccinated with 4CMenB during a 2014 campaign to control a clonal MenB outbreak.^49^ There were no cases of MenB IMD reported in vaccinated individuals in the target population after the beginning of the campaign, and no cases among 2700 unvaccinated infants who were born in 2015 after the campaign had finished, which is suggestive of herd protection.^49^ A significant vaccine effect was observed, relative risk of MenB IMD 0.22, *p* = .04,^49^ translating into an estimate of effectiveness of 78%.

Vaccine effectiveness for longer than 2 years after 4CMenB vaccination is yet to be determined. The UK infant NIP will provide important information on how immune responses correlate to protection, and the duration of protection after 4CMenB and whether a booster dose is necessary in older age groups to ensure protection throughout the childhood years when the risk of IMD remains relatively high. Preliminary data suggest only modest, if any potential for 4CMenB to induce herd protection.^53^ A UK study showed that 4CMenB vaccination of adolescents was associated with a significant decrease (26.6%, 95% CI 10.5–39.9) in the overall carriage capsular groups BCWY (mainly serogroup Y), during the first year after vaccination. An observed trend for reduced carriage of MenB strains was not statistically significant, possibly because of the low rate of MenB acquisition during the study.^53^ Strong herd protection can reduce the risk of breakthrough infections in individuals in whom immunity has waned. The potential impact of 4CMenB on carriage is being assessed in a large Australian carriage study (60,000 adolescents) currently ongoing (www.clinicaltrials.gov NCT03089086). If 4CMenB demonstrates herd protection, a combined infant and adolescent vaccination strategy would be optimal for disease control.^54^

## Conclusion

By 24–36 months after three or four doses of 4CMenB during infancy, putative seroprotective antibodies were sustained in 76% to 100% of infants for at least one 4CMenB vaccine antigen. Between 51% and 75% maintained seroprotective antibodies to either fHbp or NHBA. In toddlers, 84% to 100% maintained seroprotective hSBA concentrations for at least one 4CMenB vaccine antigen, and 59% to 75% maintained responses to fHbp or NHBA. At 4 and 7.5 years following priming during adolescence, antibody levels remained higher than at baseline and were higher than in vaccine-naïve individuals of a similar age. Marked variability amongst clinical MenB strains in the expression of 4CMenB vaccine components means that antibody levels do not necessarily translate to protection. The kinetics of the decline in vaccine-induced antibodies to 4CMenB antigens varies, with antibodies to NHBA and NadA sustained for longer than antibodies to PorA and fHbp.

Primary vaccination with 4CMenB induced robust immunologic priming, as demonstrated by rapid onset of an anamnestic response to re-vaccination. An additional booster dose induced marked rises in antibody levels to all four vaccine components. Data from NIP will help to establish correlations between the immune patterns of persistence after 4CMenB and long-term protection against IMD, as well as the eventual need and timing for booster doses.

## Trademarks

Bexsero is a trademark of the GSK group of companies. Trumenba is a trademark of Pfizer Inc.
